# Coronavirus Disease 2019 Vaccination During Pregnancy and Breastfeeding: A Review of Evidence and Current Recommendations in Europe, North America, and Australasia

**DOI:** 10.3389/fped.2022.883953

**Published:** 2022-04-29

**Authors:** Carlo Pietrasanta, Andrea Ronchi, Beatrice Letizia Crippa, Giacomo Artieri, Claudia Ballerini, Riccardo Crimi, Fabio Mosca, Lorenza Pugni

**Affiliations:** ^1^Fondazione IRCCS Ca’ Granda Ospedale Maggiore Policlinico, NICU, Milan, Italy; ^2^Department of Clinical Sciences and Community Health, Università degli Studi di Milano, Milan, Italy

**Keywords:** COVID-19, vaccine, pregnancy, breastfeeding, newborn, infant, immune response, safety

## Abstract

In the late 2020s, less than 1 year into the coronavirus disease 2019 (COVID-19) pandemic, several anti-severe acute respiratory syndrome coronavirus 2 (SARS-CoV-2) vaccines were introduced on a worldwide scale, with a significant positive impact on the consequences of the disease for several high-risk population groups. In the case of most bacterial or viral respiratory infections, pregnant women are at increased risk of complications, however, neither pregnant nor breastfeeding women were included in the first round of randomized clinical trials evaluating the safety and effectiveness of COVID-19 vaccines, because of safety and ethical concerns. Nevertheless, most anti-SARS-CoV-2 vaccines have not been expressly contraindicated during pregnancy or breastfeeding, and observational data on immune response, adverse effects, and clinical efficacy in pregnant and breastfeeding women have been progressively gathered during 2021. The vast majority of these data is reassuring for what concerns side effects for women and infants and points out the efficacy of vaccines in protecting women against COVID-19-related complications. Despite this, the hesitancy of pregnant and breastfeeding women at being vaccinated is still real. In this mini-review, we resume the available data on the clinical consequences of COVID-19 in pregnant women, as well as adverse effects, systemic and mucosal immune response, and clinical effectiveness of COVID-19 vaccines in pregnant and breastfeeding women. Moreover, we offer an updated overview of European, North American, and Australasian recommendations concerning COVID-19 vaccination in pregnant and breastfeeding women, in order to safely ensure the highest protection of women and their infants.

## Introduction

Since the end of 2019, severe acute respiratory syndrome coronavirus 2 (SARS-CoV-2) infection has been representing the greatest challenge for healthcare systems worldwide (1). Coronavirus disease 2019 (COVID-19) pandemic impacted vigorously also perinatal medicine: here, pregnant infected women present a higher risk of admission to an intensive care unit (ICU), invasive ventilation, and need for extra corporeal membrane oxygenation compared to non-pregnant reproductive-aged women, while neonates born to infected mothers, or infected themselves, can also suffer from adverse outcomes such as prematurity or respiratory distress syndrome (2, 3). The rapid introduction of anti-SARS-CoV-2 vaccines in the late 2020s has dramatically changed the trajectories of virus impact on several categories of patients, particularly the most vulnerable ones (4, 5). Pregnant women were initially excluded from clinical trials on COVID-19 vaccines, for theoretical safety and ethical concerns (6, 7). However, the progressive gathering of robust observational data from cohorts of women vaccinated during pregnancy allowed the scientific community to rapidly clarify several unresolved issues. Nevertheless, more than 1 year after the introduction of vaccines worldwide, safety concerns of pregnant or breastfeeding women are still reported as the main reason to refuse COVID-19 vaccination (8, 9), and their vaccination rates are consistently lower than those of the general population of an equivalent age (10). This attitude has been favored, to some extent, by the fact that recommendations by different national and international regulatory authorities regarding the use of anti-SARS-CoV-2 vaccines in pregnant and breastfeeding women have been repeatedly modified and amended (11). The initial (and justifiable) prudence quickly gave way to first, a more permissive and then encouraging recommendations to vaccinate both pregnant and lactating women. The vaccination offer, with country-by-country variations, was initially limited to at-risk categories such as obese, diabetic, and healthcare workers, but was rapidly extended to all pregnant and breastfeeding women.

In this “Questions and Answers” mini-review, we will explore the most updated evidence supporting the practice of COVID-19 vaccination in pregnant and breastfeeding women, and clarify major concerns that still now undermine the achievement of high vaccination rates, and summarize the current recommendations in Europe, North America, and Australasia.

At present (February 2022), 10 COVID-19 vaccines have been granted Emergency Use Listing by the WHO (complete and updated list available at https://extranet.who.int/pqweb/vaccines/vaccinescovid-19-vaccine-eul-issued), but may be approved or not for clinical use in different countries. For reasons of convenience, this review will be focused on data and recommendations existing for two mRNA vaccines (Comirnaty by Pfizer BioNTech Manufacturing GmbH, and Spikevax by Moderna Biotech), two non-replicating viral vector vaccines (Vaxzevria by Astrazeneca AB, and Ad26.COV2-S [recombinant] by J and J/Janssen), and one recombinant protein subunit (Spike) vaccine (Nuvaxovid by Novavax).

## Is Anti-severe acute respiratory syndrome coronavirus 2 (SARS-CoV-2) Vaccination Useful During Pregnancy?

The answer to this question can now be grounded in robust scientific data: a large meta-analysis (12) by a British research team collected available data on over 64,000 pregnant women infected with SARS-CoV-2, showing that pregnant women with infection have a significantly higher risk (unadjusted for confounders) of hospitalization in the ICU (OR: 2.13, 95% CI: 1.53–2.95), of mechanical ventilation (OR: 2.59, 95% CI: 2.28–2.94), and of extracorporeal circulation (OR: 2.02, 95% CI: 1.22–3.34) compared to infected, non-pregnant women. In addition, infection during pregnancy increases the risk of maternal death, preeclampsia, premature birth, and intrauterine death, with odds ratios ramping up as the severity of maternal disease increases (13). Compared to the amount of data available for pregnant women, those regarding SARS-CoV-2 infection in neonates are significantly more limited and less robust. Vertical transmission rates of SARS-CoV-2 from an infected mother to her fetus during pregnancy are currently estimated at around 2–3%, based on a neonatal screening strategy consisting solely of rt-PCR for SARS-CoV-2 RNA on nasopharyngeal swabs (14, 15). After the WHO enacted more accurate definitions for confirmed, probable, or unlikely vertical transmission (16), including longitudinal analysis of multiple sterile and non-sterile body sites, the precise vertical transmission rate of COVID-19 remains to be established in large cohorts. Nevertheless, the impact of SARS-CoV-2 on neonates is not limited to the vertical transmission from an infected mother: indeed, data from the Swedish neonatal registry clearly showed that maternal infection during pregnancy can worsen neonatal outcomes independently of the vertical transmission of the virus. Neonates born to mothers with perinatal COVID-19 present an increased risk of resuscitation in the delivery room, mechanical ventilation, persistent pulmonary hypertension, and of jaundice requiring treatment compared to neonates born to unaffected women (3).

Moreover, neonates can acquire SARS-CoV-2 infection from their mothers even after birth, through a horizontal airborne transmission that seems favored by severe maternal COVID-19, possibly sustained by a high viral load (17). Most of these data were collected in 2020 or early 2021, during the first two waves of the pandemic, when the original strain of the virus or variants whose severity has been later de-escalated, such as the Alpha (B.1.1.7), were prevalent in Europe, North America, and Australasia. In late 2021, limited but worrying data regarding the impact of the Delta (B.1.617.2) variant on pregnant women raised significant concern among healthcare providers, as it was clearly shown that Delta infection during pregnancy significantly increased the proportion of severe or critical disease (36 vs. 13%, aRR: 2.76, 95% CI: 1.73–4.40) and ICU admissions (29 vs. 8%, aRR: 3.42, 95% CI: 1.91–6.11) compared to the pre-Delta period (18). In slightly more than 2 months, the Omicron variant (B.1.1.529) then blew away the Delta all over the world throughout the winter of 2021–2022. The impact of Omicron on perinatal medicine is yet to be established, and the interpretation of data after 2 years of pandemic and the introduction of COVID-19 vaccination may be confused by pre-existing natural or vaccine-induced immunity (19). However, it is nowadays clear that safe and effective anti-SARS-CoV-2 vaccines administered to pregnant women might greatly reduce the negative impact of COVID-19 on both pregnant women and newborns (20), as will be discussed more in detail in the next sections.

## Is Vaccination During Pregnancy Safe for the Mother and the Infant?

Safety concerns are still reported as the main reason to refuse COVID-19 vaccination during pregnancy (21, 22). The hesitancy of pregnant women to receive vaccines that have been developed faster than any other in history, and for which there is no long-term safety data, is more than justifiable. Therefore clear, evidence-based data are necessary to promote the highest possible adherence to current recommendations. Until February 2022, the US-based “V-safe” register collected self-reported data from over 198,000 women who were pregnant at the moment of COVID-19 vaccination (23). Similarly, more than 100,000 women were reported to have received the COVID-19 vaccine in the United Kingdom (24). These data are constantly updated and have not raised concerns regarding possible serious adverse events caused by or strictly related to vaccination. In particular, the administration of mRNA vaccines (Comirnaty by Pfizer—BioNTech and Spikevax by Moderna) is not associated with a higher incidence of prematurity, with the delivery of neonates small for gestational age (SGA), nor with increased proportions of congenital malformations compared to the standard incidence in non-vaccinated women (25). Furthermore, two large US-based observational studies demonstrated that the cumulative risk of spontaneous abortion, from conception to the 19th week of pregnancy, after COVID-19 vaccination during the first trimester was 14.1% (CI: 12.1–16.1%), in line with that of historical cohorts of unvaccinated women (26, 27). The risk remained stable regardless of the week of administration of the first dose. Recently, data from two large population-based observational retrospective cohorts evaluating outcomes in more than 250,000 pregnancies from Canada, Sweden and Norway were extremely reassuring, especially for pregnant women vaccinated with mRNA vaccines in the second or third trimester of pregnancy. Specifically, in the cohort study conducted in Ontario, Fell et al. reported that COVID-19 vaccination during pregnancy was not significantly associated with increased risk of postpartum hemorrhage, chorioamnionitis, cesarean delivery, admission to neonatal intensive care unit, or low Apgar score compared to vaccination after pregnancy and to no vaccination, even when adjusted for confounding factors (28). Similarly, data from Scandinavian registries showed how vaccination during pregnancy was not significantly associated with increased risk of preterm birth, stillbirth, small gestational age, low Apgar score, or neonatal admission to intensive care unit (29).

Like any other individual, pregnant women can suffer from post-injection side effects, which are more common after the second or third vaccine dose compared to the first for mRNA vaccines, and apparently more frequent after the first dose for adenovirus (Ad) vector-based vaccines (30). However, the reported rates of side effects after receiving mRNA COVID-19 vaccines do not significantly differ from those of non-pregnant women (25). Among post-injection side effects, fever after the second dose is reported by 46% of women after the Moderna vaccine and by 24.8% after the Pfizer vaccine (25). As maternal fever during the first trimester can be associated with an increased relative risk of congenital malformation (e.g., cleft lip and neural tube closure defects) (31), some national regulatory agencies such as the Italian Ministry of Health recommended a case-by-case evaluation before the administration of COVID-19 vaccines during the first trimester of pregnancy and endorsed a full recommendation only for vaccination in the second and third trimester. However, considering the risks associated with COVID-19 during pregnancy, other authorities such as the Royal College of Obstetricians and Gynecologists (United Kingdom), the American College of Obstetricians and Gynecologists (united states), the Society of Obstetrician and Gynecology of Canada, the Royal Australian and New Zealand College of Obstetricians and Gynecologists opted anyway for an “as early as possible” advice, recommending pregnant women to receive vaccination (first, second, or booster dose) at any time during pregnancy.

Current national recommendations from selected regulatory agencies worldwide are summarized in Table 1. Safety data concerning the vaccination of pregnant women with the non-replicating viral vector vaccines Janssen (Ad26.COV2-S, J&J/Janssen) and VaxVeria (ChAdOx1-S, AstraZeneca), and with the S-protein recombinant adjuvanted Nuvaxovid (Novavax) are greatly limited compared to those available for mRNA vaccines. In non-pregnant adults, both Ad26.COV2-S and Nuvaxovid are associated with slightly lower rates of post-injection side effects compared to both Moderna and Pfizer mRNA vaccines (32, 33), while animal studies have shown no clear adverse effect of vaccination on pregnancy or neonatal outcomes. However, observational data on pregnant women are limited and do not enable most regulatory agencies to fully recommend their use during pregnancy. Thrombosis with thrombocytopenia syndrome (TTS) is a serious but extremely rare condition occurring in approximately 1–2/100,000 doses of Janssen or VaxVeria vaccine administered to females aged 30–39 years (34). A warning about the possibility of TTS occurrence after administration of ad vector-based COVID-19 vaccines has been included in the Emergency Use Authorization (EUA) of most countries. The use of ad vector-based COVID-19 vaccines (Janssen in the US, Janssen, and VaxVeria in the EU and United Kingdom), as well as of Nuvaxovid in pregnant women is currently allowed worldwide, but all regulatory agencies state that mRNA vaccine should be routinely preferred unless a clear contraindication or a strong preference of the patient exists (see Table 1). To date, there is no evidence that pregnant or postpartum women are at higher risk of vaccine-induced TTS than non-pregnant age-matched women (35). However, considering that pregnancy itself increases the risk of thrombosis four to fivefold (36), the risk of TTS in pregnant women who receive Janssen or VaxVeria vaccines deserves further evaluation.

**TABLE 1:** National and supranational selected recommendations regarding coronavirus disease 2019 (COVID-19) vaccination during pregnancy and breastfeeding.

Location	Vaccines approved (EUA*) for pregnant women	Vaccines preferred for pregnant women	Timing of vaccination	Booster dose
EU**^a^	- Pfizer-BioNTech: BNT162b2 - Moderna: mRNA-1273 - Janssen: Ad26.COV2.S - AstraZeneca: ChAdOx1-S - Novavax: NVX-CoV2373	- Pfizer-BioNTech: BNT162b2 - Moderna: mRNA-1273	- Before pregnancy (any time) - During pregnancy (any trimester**) - After pregnancy (any time)	No specific recommendation
**UK*^b^***	- Pfizer-BioNTech: BNT162b2 - Moderna: mRNA-1273 - Janssen: Ad26.COV2.S - AstraZeneca: ChAdOx1-S	- Pfizer-BioNTech: BNT162b2 - Moderna: mRNA-1273	- Before pregnancy (any time) - During pregnancy (any trimester) - After pregnancy (any time)	Yes, at any time. mRNA vaccines preferred (even after non-replicating viral vector vaccine cycle)
**US*^c^***	- Pfizer-BioNTech: BNT162b2 - Moderna: mRNA-1273 - Janssen: Ad26.COV2.S	- Pfizer-BioNTech: BNT162b2 - Moderna: mRNA-1273	- Before pregnancy (any time) - During pregnancy (any trimester) - After pregnancy (any time)	Yes, at any time. mRNA vaccines preferred (even after non-replicating viral vector vaccine cycle)
**Canada*^d^***	- Pfizer-BioNTech: BNT162b2 - Moderna: mRNA-1273 - Janssen: Ad26.COV2.S - AstraZeneca: ChAdOx1-S - Novavax: NVX-CoV2373 - Medicago-GSK: CoVLP	- Pfizer-BioNTech: BNT162b2 - Moderna: mRNA-1273	Before pregnancy (any time) - During pregnancy (any trimester) - After pregnancy (any time)	Yes, at any time.
**Australia and NZ*^e^***	- Pfizer-BioNTech: BNT162b2 - Moderna: mRNA-1273 - AstraZeneca: ChAdOx1-S	- Pfizer-BioNTech: BNT162b2 - Moderna: mRNA-1273	- Before pregnancy (any time) - During pregnancy (any trimester) - After pregnancy (any time)	Yes, at any time. mRNA vaccines preferred (even after non-replicating viral vector vaccine cycle)

*Emergency Use Authorization. **Country-specific differences may exist (e.g., Italy: vaccination during the second and third trimester of pregnancy is recommended. For vaccination during the first trimester a cos-benefit analysis is encouraged). Online references, accessed March 15, 2022:

*^a^https://www.ema.europa.eu/en/news/covid-19-latest-safety-data-provide-reassurance-about-use-mrna-vaccines-during-pregnancy.*

*^b^https://www.rcog.org.uk/media/kbknl3z3/2022-03-07-coronavirus-covid-19-infection-in-pregnancy-v15.pdf.*

*^c^https://www.cdc.gov/coronavirus/2019-ncov/need-extra-precautions/pregnant-people.html; https://www.acog.org/-/media/project/acog/acogorg/files/pdfs/clinical-guidance/practice-advisory/covid19vaccine-conversationguide-121520-v2.pdf*

*^d^https://sogc.org/common/Uploaded%20files/Latest%20News/SOGC_Statement_COVID-19_Vaccination_in_Pregnancy.pdf.*

*^e^https://ranzcog.edu.au/statements-guidelines/covid-19-statement/covid-19-vaccination-information.*

## Is Vaccination During Pregnancy Effective for the Mother and the Infant?

COVID-19 vaccination of pregnant and breastfeeding women has been shown to induce humoral and cell-mediated responses akin to that induced in young non-pregnant women (37). Vaccination-induced serum antibodies, both anti-Spike (anti-S) and anti-Receptor-Binding Domain (RBD), are mainly of the IgG class and persist for at least 6–9 months after maternal vaccination (38). Pending the results of randomized trials conducted on pregnant women, observational data from two large Israeli cohorts have shown a significant decrease in COVID-19 cases among pregnant women after vaccination, and overall efficacy of around 96% (CI: 89–100%) for confirmed infection, of 97% (CI: 91–100%) for symptomatic confirmed infection and of 89% (CI: 43–100%) for hospitalization due to COVID-19, starting from 7 days after the administration of the second dose (39, 40). These data mainly reflect the effectiveness against B1.1.7 (Alpha) variant and the original SARS-CoV-2 strain, which were dominant in Israel during the study period. During the last trimester of pregnancy, vaccine-induced IgG is actively transferred to the fetus *via* the placenta through Fc receptors. The maternal serum/cord transfer ratio positively correlates with the distance between the end of the maternal vaccination cycle and the time of delivery and can exceed 1 (41, 42). It is now clear that anti-S and anti-RBD IgG induced by maternal vaccination during pregnancy can persist in neonatal serum up to at least 6 months, a time span much longer than that of antibodies induced by maternal natural infection (43), while the effective clinical protection against COVID-19 hospitalization conferred to infants aged < 6 months has been recently estimated for the first time, in a case-control study, and seems equal to 61% (95% CI: 31–78%) (44). COVID-19 vaccination during pregnancy also induces the production of IgG, IgA, and IgM antibodies in breast milk, although in much lower quantities than in serum and probably not persistent for such a long time (45, 46). Indeed, akin to what occurs for several other vaccines administered intramuscularly (47), the activation of mucosal plasma cells of the mammary gland induced by COVID-19 vaccines may be limited, as highlighted by the relative low (compared to IgG) amount of SARS-CoV-2—specific IgA recovered in the breast milk of vaccinated women (46). Whether breast milk vaccine-induced antibodies may confer some degree of protection to breastfed infants is certainly conceivable, but not proven yet.

## Is Vaccination During Breastfeeding Safe and Effective for the Mother and the Infant?

Available scientific evidence and recommendation by the included regulatory agencies worldwide do not contraindicate the administration of COVID-19 vaccines (either first, second, or booster dose) during breastfeeding (Table 1). For breastfeeding mothers, the clinical efficacy of the COVID-19 vaccine is not expected to be different from other women, although data from observational cohorts or randomized control trials are still pending. Moreover, lactating women are encouraged not to interrupt breastfeeding before or after vaccination. For lactating mothers, indeed, the expected side effects are the same as those found in non-lactating women, and even in this population, they occur with a slightly higher incidence for the Moderna vaccine than for Pfizer (48). During the 48–72 h after vaccination, breastfeeding women may experience a transient reduction in milk production, a side effect that appears to occur more frequently after the second dose of vaccine, and to a greater extent (23.4% of women) for the Moderna vaccine than for Pfizer (8%) (48). No significant side effect (fever, rash, cough, behavioral change, vomiting, or diarrhea) has been recorded in infants breastfed by vaccinated women within 72 h after BNT162b2 vaccination as recently reported in a prospective study (49). Importantly, there is no clear evidence that mRNA-based vaccines do not significantly diffuse to breast milk, where the vaccine mRNA sequence has not been detected (50, 51), or has been detected in minimal amounts, with no presumable biological activity and high susceptibility to rapid enzymatic degradation (49). Maternal vaccination during breastfeeding is effective in inducing SARS-CoV-2 specific serum antibodies of IgG, IgM, and IgA class in the mother, despite delayed kinetics of antibody titers and FcR-binding capacity compared to non-pregnant or non-lactating ones (45, 46). Specific and neutralizing antibodies are also induced in breast milk, albeit in significantly lower amounts as compared to serum concentrations and, specifically for IgA, in lower amounts as compared to natural infection (37). Akin to maternal vaccination during pregnancy, the capacity of breast milk antibodies induced by maternal vaccination during breastfeeding to confer some mucosal protection against SARS-CoV-2 infection to breastfed infants is yet to be established.

## Is Vaccination During Pregnancy and Breastfeeding Also Effective Against the Delta and Omicron Variants?

Most data concerning the immunological and clinical efficacy of COVID-19 vaccines in pregnant and lactating women were obtained in the “pre-Delta” period, in epidemiological contexts dominated by viral variants, such as the Alpha (B.1.1.7), the Beta (B.1.351), or the Gamma (P.1) variant in South America, that are not prevailing anymore. Between May and December 2021, the Delta variant (B.1.617.2) became predominant in Europe, North America, and Australasia, while since the end of 2021 the newly emerged Omicron (B.1.1.529) has been responsible for more than 90% of COVID-19 cases in the same areas (52). Both the Delta and the Omicron variants were classified by the WHO as “variants of concern” (VOC), because of their increased contagiousness and/or virulence compared to both the original SARS-CoV-2 strain and most previous variants. SARS-CoV-2 genome is extremely prone to the acquisition of new mutations, which are also favored by the velocity of virus diffusion in the twenty-first century. Consequently, the global epidemiological scenario changes continuously, imposing a non-stop reassessment of previously acquired data. The first available evidence suggested that the Delta variant might worsen the outcomes of the infected pregnant woman compared to other viral variants, increasing the rate of severe infections, the need for hospitalization, and, as reported in a small case series, the probability of placentitis and fetal demise (53, 54). The short period of Omicron prevalence has not allowed yet to collect robust epidemiological data for pregnant and lactating women. For what concerns vaccine efficacy, data on women of childbearing age have recently been published from the United Kingdom, where over 380,000 individuals over the age of 18 have been vaccinated in an epidemiological setting dominated by the Delta variant (55). The efficacy of the Pfizer vaccine was reduced against a symptomatic or high viral load (Ct < 30) Delta variant infections compared to the Alpha strain of SARS-CoV-2, but was still equal to 84% (95% CI: 82–86%). Further analysis also revealed that vaccine effectiveness tended to wane by about 22% every 30 days after the receipt of the second dose. For what concerns the efficacy of vaccines administered during pregnancy against the Omicron variant, the first available data suggest that the neutralizing capability of serum from vaccinated pregnant women might be lower for the Omicron variant as compared to wild type SARS-CoV-2, or Beta and Delta variants (56). However, another recent report confirmed a reduced RBD recognition and Fc-receptor binding against the Omicron variant but highlighted the preservation of Omicron Spike-specific antibodies, that may continue to attenuate disease severity in pregnant women (57). Translating this evidence into the clinical scenario is not easy, and data are still scarce: the first available report was recently published as a Morbidity and Mortality Weekly Report (MMWR) by the Centers for Disease Control and Prevention (CDC): it is a case-control study on infant protection after maternal vaccination, reporting effectiveness of maternal vaccination during pregnancy against COVID-19 Delta and Omicron hospitalization in infants aged < 6 months of 61% (95% CI: 31–78%) (44). Further data on the effectiveness of maternal outcomes are yet to be collected. Finally, there is currently no available evidence regarding the effectiveness of vaccines in breastfeeding women against VOCs; however, for women, this is reasonably expected not to be different from that of non-breastfeeding women in their childbearing age. Conversely, it has been recently shown that the efficacy of breast milk antibodies (mainly IgA) in binding to most VOCs, including the Delta variant, was reduced compared to the original Wuhan-Hu-1 strain, possibly highlighting reduced clinical protection of breastfed neonates after maternal vaccination during breastfeeding (58).

## Conclusion

The administration of COVID-19 vaccines to pregnant or breastfeeding women is safe in the vast majority of cases and is proving effective in reducing adverse consequences of SARS-CoV-2 infection for mothers and their infants. Every effort should be made by national and supranational authorities to provide clear and harmonized recommendations to pregnant and breastfeeding women, in order to achieve the highest possible immunization coverage in this high-risk population group.

## Author Contributions

CP, AR, FM, and LP conceived the article. CP, AR, BC, GA, CB, and RC revised literature and recommendations. CP wrote the first draft. All authors revised the draft, provided insightful contributions, and agreed to be accountable for the content of the work.

## Conflict of Interest

The authors declare that the research was conducted in the absence of any commercial or financial relationships that could be construed as a potential conflict of interest.

## Publisher’s Note

All claims expressed in this article are solely those of the authors and do not necessarily represent those of their affiliated organizations, or those of the publisher, the editors and the reviewers. Any product that may be evaluated in this article, or claim that may be made by its manufacturer, is not guaranteed or endorsed by the publisher.
